# Minimal seasonal variation in disease parameters of axial spondyloarthritis: a register-based study

**DOI:** 10.1007/s00296-025-05913-4

**Published:** 2025-07-17

**Authors:** Emma Onkamo, Björn Sundström, Bengt Wahlin

**Affiliations:** 1 Department of Public Health and Clinical Medicine/Rheumatology, Umeå University, Umeå, 901 87 Sweden; 2 Centre for Research and Development, Uppsala University/Region Gävleborg, Gävle, 801 88 Sweden; 3 Department of Public Health and Caring Sciences/General practice, Uppsala University, Uppsala, 751 22 Sweden

**Keywords:** Axial spondyloarthritis, Seasons, Patient reported outcome measures, Inflammation

## Abstract

**Objectives:**

Axial spondyloarthritis (axSpA) displays varying disease activity over time. However, few studies have examined the association between disease activity and seasonal differences, and consequently, evidence of a seasonality in disease activity in axSpA is limited. We therefore aimed to study the variation in measures of disease activity, perceived well-being, and functional ability, including both patient-reported and objective measures throughout the year, and their relationship with seasons.

**Methods:**

Objective and patient-reported disease parameters from 44 987 assessments on 5197 individuals were retrieved from the Swedish Rheumatology Quality register. The variation in different measures of disease activity over the year was examined using generalized additive models with cyclic cubic splines.

**Results:**

Patient reported and objective measures of disease were found to vary congruently over the year, with a trend of decreased disease parameters in the spring. Mean CRP values showed a statistically significant variation throughout the year, with highest in July (7.35 mg/L) and lowest in March (6.88 mg/L). Significant seasonal variation was also seen in BASDAI (range of mean values 3.67–3.72), BASFI (2.79–2.87) and BASMI (3.26–3.31), with highest values in late summer, beginning of fall, or in the fall, respectively. In subgroup analysis, significant variation was only seen in subjects with a disease duration of more than 10 years.

**Conclusion:**

Although a statistically significant seasonal variation in disease parameters was found, the absolute variation was close to none and may therefore be clinically irrelevant.

**Supplementary Information:**

The online version contains supplementary material available at 10.1007/s00296-025-05913-4.

## Introduction

Axial spondyloarthritis (axSpA) is a chronic inflammatory disease of unknown etiology, mainly affecting sacroiliac joints and the vertebral column, causing pain and stiffness. Extra-axial manifestations include peripheral arthritis, enthesitis and acute uveitis [1]. Disease onset typically occurs before the age of 30, and men are more frequently affected than women [2].

Axial spondyloarthritis is characterized by a variation in disease activity over time, as seen in other inflammatory diseases, such as rheumatoid arthritis (RA) [3]. Moreover, it is common that patients with inflammatory diseases, including axSpA, describe variations in disease activity which they associate with changes in weather and season. This is however insufficiently studied, and no studies of this subject have been performed in Sweden. The few studies regarding how disease activity in inflammatory joint diseases varies with weather and season have mainly focused on patients with RA [4–6]. For AS, studies assessing the relationship between weather, seasonal changes and variation in disease activity are even fewer, with just three published studies upon small populations [7–9]. No conclusions can be drawn from these studies, due to inconclusive results and the usage of different measures to examine disease activity. A Canadian study of weather and disease activity in psoriatic arthritis could not identify any difference in measures of axial morbidity between summer and winter [10].

Disease activity and physical function in axSpA is commonly assessed using standardized and validated measuring instruments. The most frequently used are Bath Ankylosing Spondylitis Disease Activity Index (BASDAI) and Bath Ankylosing Spondylitis Functional Index (BASFI), which are based entirely on patient-reported outcome measures [11, 12]. Also, objective markers of inflammation, such as plasma levels of C-reactive protein (CRP) and erythrocyte sedimentation rate (ESR), are commonly used to assess disease activity. These biomarkers are also integrated with patient reported outcome measures in Ankylosing Spondylitis Disease Activity Score (ASDAS) [13]. To objectively assess spinal mobility, Bath Ankylosing Spondylitis Metrology Index (BASMI) is used [14].

As mentioned above, it is unclear to what extent disease activity in axSpA varies with seasons. Seasonal variation of disease activity, if present, could however confound evaluation of pharmacological treatment and measures of rehabilitation, and thus this issue should be evaluated further. The aim of this study was therefore to determine if measures of disease activity, physical function and well-being vary over the year, and whether these variations are associated with season. A further aim, if such variation exists, was to study whether objective measures (CRP, ESR, and axial mobility) and patient-reported measures are congruent, or if there are seasonal discrepancies.

## Materials and methods

### Data collection

Assessments of disease from 5197 individuals with axSpA registered at 44 987 time points from February 2001 to March 2022, were acquired from the Swedish Rheumatology Quality register (SRQ). Patients included in SRQ are mainly treated in specialist clinics, while patients with limited disease treated in the primary care are usually not included in SRQ. In the present study, individuals in SRQ above the age of 18, diagnosed with ankylosing spondylitis (ICD code M45.9) with registered assessments in both patient reported measurements (BASDAI, BASFI, BAS-G) and objective measures (BASMI, CRP) were included. The accuracy of diagnoses in this register have previously been validated [15]. Register data for each assessment included measures of disease activity, current treatment and reported symptoms, as well as location and date for patient visits. This study was approved by the Swedish Ethical Review Authority 2021-12-13 and 2023-03-29 (2021-06234-01, 2023-01798-02). Participant consent was not obtained as this was a register-based study with anonymized data.

### Measures of disease

Disease activity, physical function and perceived well-being was measured through registered data regarding CRP and ESR as well as scoring from standardized evaluation tools. These tools were BASDAI, and ASDAS for assessing disease activity, BASFI and BASMI for assessing physical function, and Bath Ankylosing Spondylitis Global Score (BAS-G) and Eq. 5D for assessing global well-being and quality of life, respectively. All these indices have been described and validated elsewhere [11–14, 16, 17]. For subgroup analysis, the previously established minimums for clinically relevant differences between assessments of BASDAI (1 cm), BASFI (0.7 cm) and BASMI (30%) were used [14, 18]. Disease duration was defined as time elapsed from the onset of first symptom to time of each individual assessment. To analyze the impact of summer holidays, where patients with more acute disease manifestations are prioritized, this was examined separately. The summer holidays generally start around midsummers eve, late June, and continues throughout July and the first two weeks of August.

### Definition of seasons in geographical areas

To define seasons, we have used the Swedish Meteorological and Hydrological Institute’s (SMHI) criteria, which is based on daily mean temperature. According to these criteria, a new season starts after five consecutive days of mean temperatures characteristic of that season, except for spring, which requires seven consecutive days. Winter is defined as temperatures below 0 °C and summer as temperatures above 10 °C, while spring and fall temperatures are defined as temperatures in the range of 0 °C and 10 °C [19].

Data on the average dates for the start of seasons for the years 1991–2020 in Sweden was collected from SMHI. Counties with similar dates of seasonal change were grouped together, dividing the 21 counties in Sweden into three geographical areas: northern, middle, and southern Sweden. These geographical areas differ in climate and seasonal variation. The winters are long, cold, and snowy in northern Sweden, whereas, in southern Sweden, winters are short with an average temperature close to 0 °C. The difference in weather and temperature between the three regions is smaller during the other seasons, making variation in temperature between seasons larger in northern Sweden.

### Statistical analysis

The variation in disease parameters throughout the year was examined using generalized additive models with cyclic cubic splines to account for seasonal variations within each region and across the entire country, as well as a random intercept for individual patients. Values of CRP higher than 100 mg/L were excluded, as well as registered values of BASMI, BASDAI and BASFI above 10. Student’s T-test was performed to compare means, Bonferroni adjusted when appropriate. The statistical analyses were performed using the gamm4 package in RStudio version 4.3.2. All significance levels were set at *p* < 0.05.

## Results

**Fig. 1:**
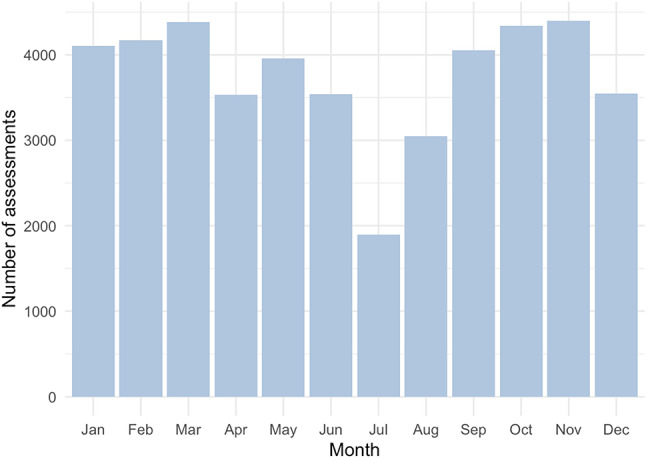
Distribution throughout the year of assessments of disease activity in individuals with ankylosing spondylitis during the period 2001–2022

The material contained in total 44,987 assessments of disease parameters, including BASDAI (*n* = 27 877), BASFI (*n* = 25 656) as well as BASMI (*n* = 7539), Eq. 5D (*n* = 18 596) and CRP (*n* = 32 699). The material also contained measurements of ESR (*n* = 30 844), ASDAS ESR (*n* = 18 100), ASDAS CRP (*n* = 19 242), along with measurements of BAS-G (one week, *n* = 21 864), and BAS-G (six months, *n* = 21 862). Demographic data on the 5197 participants is presented in Table 1. The number of patient assessments per month showed notably lower during summer months, especially in July (Fig. 1).

**Table 1:** Demographic data on the 5197 individuals with ankylosing spondylitis included in a study regarding seasonal variations in disease parameters

Male, *n* (%)	3530 (68)
Age at inclusion, years	44 (14)
Disease duration at inclusion, years	16 (13)
Number of assessments, median (IQR)	7 (3–12)
Follow-up time, years	5.4 (4.3)
Biological treatment ever, n (%)	3968 (76)
Ever smoking (*n* = 4740), n (%)	2522 (53)

Numbers are presented as mean (SD), except when indicated else

**Fig. 2:**
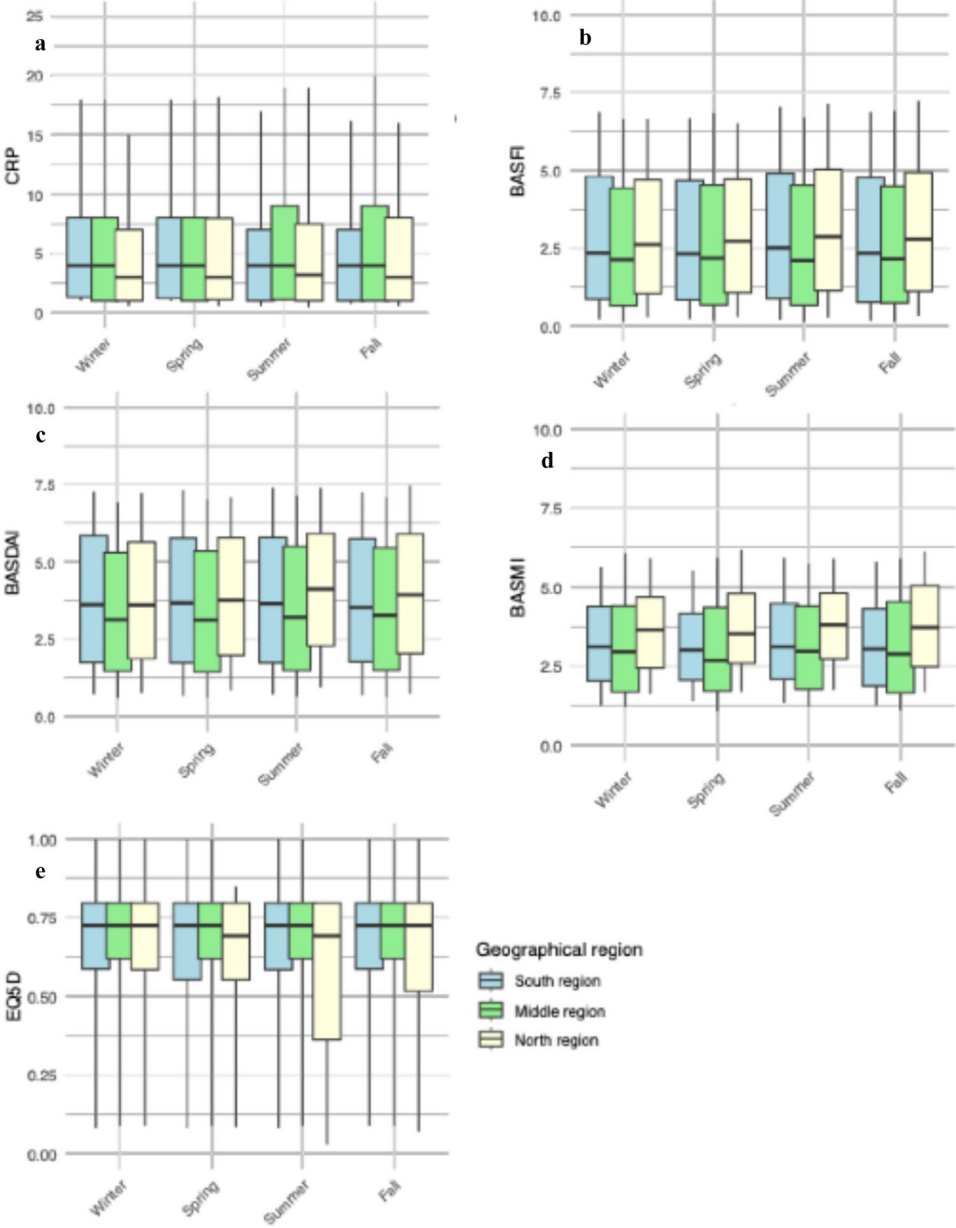
Box plots displaying distribution of CRP (**a**), BASFI (**b**), BASDAI (**c**), BASMI (**d**) and EQ5D (e) in individuals with ankylosing spondylitis, split by seasons and regions. The boxes represent the interquartile range, with the line inside indicating the median. The whiskers extend to the 10th and 90th percentiles.* BASDAI*: Bath Ankylosing Spondylitis Disease Activity Index.* BASFI:* Bath Ankylosing Spondylitis Functional Index.* BASMI:* Bath Ankylosing Spondylitis Metrology Index.* CRP:* C-reactive protein.

**Fig. 3:**
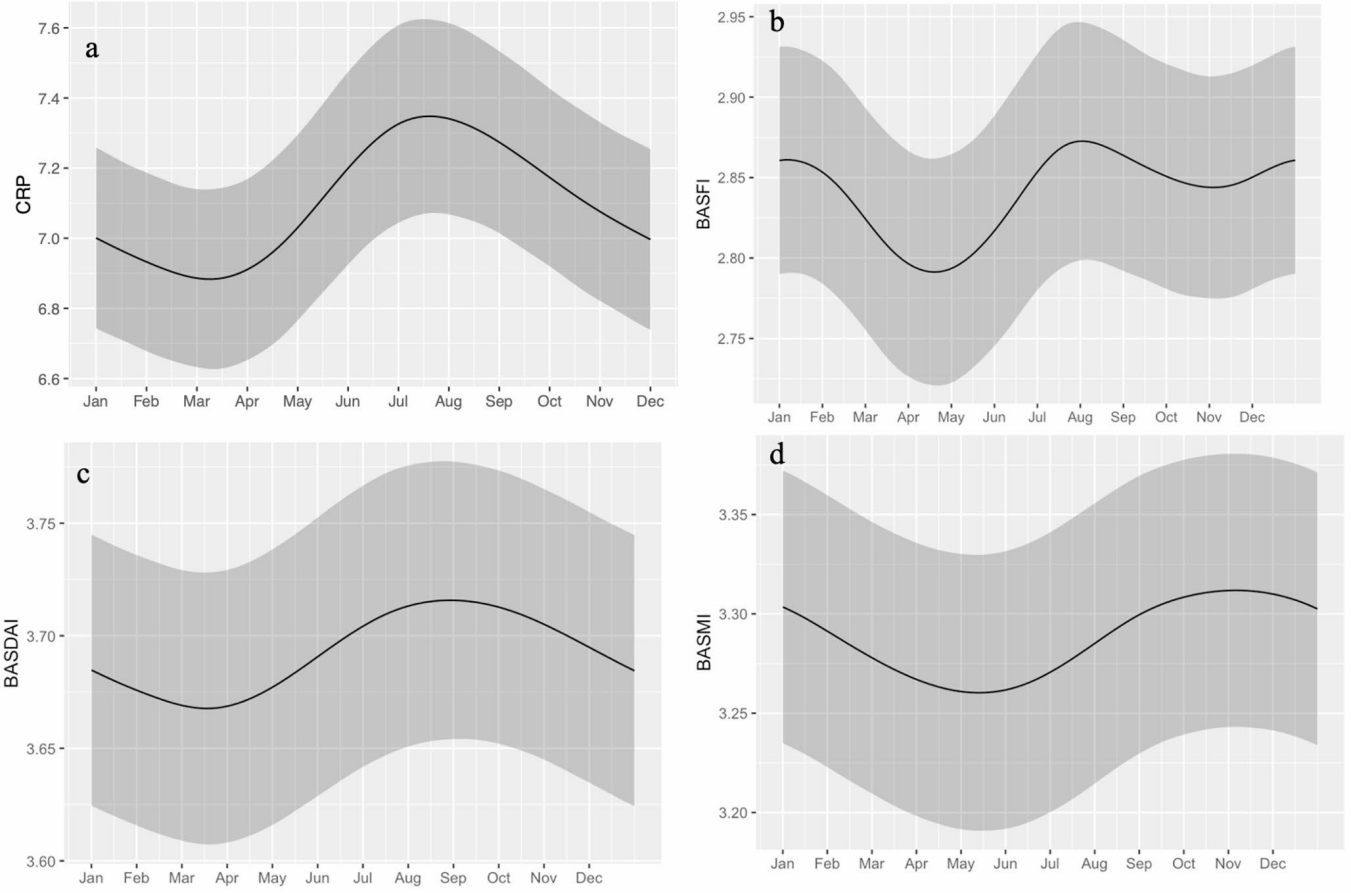
Temporal variation, for the entire country, in mean values with splines. Figure** a** displaying CRP (*p* < 0.001), figure** b** displaying BASFI (*p* < 0.01), figure** c** displaying BASDAI (*p* < 0.05) and figure** d** displaying BASMI (*p* < 0.01). The main line represents the smoothed mean curve, while the ribbon signifies the 95% confidence interval. *BASDAI*: Bath Ankylosing Spondylitis Disease Activity Index. *BASFI*: Bath Ankylosing Spondylitis Functional Index. *BASMI*: Bath Ankylosing Spondylitis Metrology Index. *CRP*: C-reactive protein.

Mean values of BASDAI, BASFI, BASMI, CRP and EQ5D split by seasons and regions, illustrating small regional differences (Fig. 2). When analyzing the variation in disease parameters over the year in the entire population, mean values of CRP varied significantly with peak values during summer (Fig. 3A), with a range between 7.35 and 6.88 mg/L. Both BASDAI and BASFI varied statistically significantly, with the highest mean values reported in the late summer or beginning of fall (Fig. 3B and C). The maximum and minimum mean values ranged between 3.72 and 3.67, for BASDAI, and 2.87 and 2.79 for BASFI. Also, BASMI displayed a significant temporal variation (Fig. 3D) with a range between 3.31 and 3.26, where the highest values were found in the fall, after the peaks in CRP, BASDAI and BASFI.

**Fig. 4:**
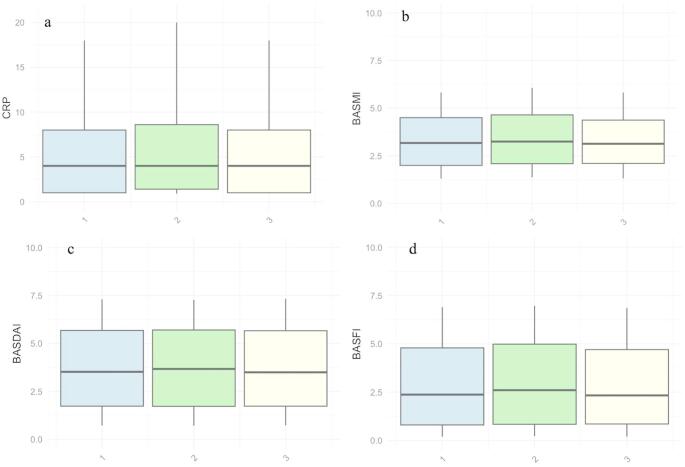
Box plots displaying distribution of CRP (**a**), BASFI (**b**), BASDAI (**c**), BASMI (**d**) split by three different periods. Group 1 represents one month before Midsummer’s Eve, group two represents the period from Midsummer’s Eve and August 15th, while the third group represents August 16th to September 15th, i.e. the time before, during and after the summer holidays. The boxes represent the interquartile range, with the line inside indicating the median. The whiskers extend to the 10th and 90th percentiles. *BASDAI*: Bath Ankylosing Spondylitis Disease Activity Index. *BASFI*: Bath Ankylosing Spondylitis Functional Index. *BASMI*: Bath Ankylosing Spondylitis Metrology index. *CRP*: C-reactive protein.

Peak mean values of BASDAI (3.84), BASFI (3.12), BASMI (3.51) and CRP (8.17 mg/L) were all found during the period of summer holiday (Fig. 4). However, mean values of BASDAI, BASFI, and BASMI were not significantly higher than during the time periods before or after summer holidays. Regarding mean CRP, the mean values was significantly higher than before summer holidays (*p* = 0.02), but after Bonferroni adjustment the difference was not significant.

Regarding EQ5D, BAS-G, ESR and ASDAS, no statistically significant seasonal variation was found. However, the variation throughout the year followed the same pattern as other measures (supplementary material, Fig. 1). Similar results were found when examining different subgroups within our population. Thus, the seasonal variation was equal in men and women (supplementary material, Fig. 2).

In individuals with a clinically relevant difference between highest and lowest values of BASDAI, BASFI or BASMI, the seasonal variation followed the same pattern as the variation observed in the entire study population (Supplementary material, Fig. 3). For BASDAI, the maximum and minimum mean values were 3.80 and 3.76 respectively. For BASFI they were 3.12 and 3.04, and BASMI they were 2.49 and 2.38.

No subgroup based on disease duration (< 10 years vs. >10 years) showed larger seasonal variation in disease parameters. In individuals with a disease duration of less than 10 years (12 051 assessments), no statistically significant seasonal variation was found. The group with a disease duration of more than 10 years (32 565 assessments) showed a statistically significant variation throughout the year, like the variation observed in the entire study population (data not shown).

When analyzing the individual geographical regions, no region displayed a larger seasonal variation than the variation observed when analyzing data from the entire country (Fig. 2).

## Discussion

In this large Swedish register study on over 44 000 registered assessments of disease activity, physical function and well-being in individuals with axSpA, we found a statistically significant seasonal variation in disease parameters. During summer and early fall higher disease activity was observed, with a trend of decreased disease activity during spring. Absolute seasonal variation in disease activity and mobility was however minimal.

It is unclear whether our results represent a true variation in disease activity or if it is an effect of prioritizing patients with a more acute disease manifestation and higher disease activity during the summer holidays and the time thereafter, thus making the registered values of disease activity higher. When comparing the mean values of CRP, BASDAI, BASFI and BASMI, before, during and after the period of summer holidays no statistically significant variation was found. However, for all the variables examined, the mean values during the summer holidays were numerically larger compared to the maximum mean values seen in the models of disease parameters over the year. This may indicate that the, seemingly, increased disease activity during summer months may be due to organizational factors rather than an actual increase in disease activity.

Regarding analyses of individuals with a clinically significant variation between the highest and lowest value in BASDAI, BASFI and BASMI no statistically significant variation throughout the year was found, the pattern of seasonal variation was like the pattern seen in the entire study population. The amplitude was slightly larger in BASMI values, this observed difference is still clinically insignificant.

Our study showed a statistically significant variation in mean CRP values, with the highest values during summer. To the best of our knowledge, only one previous study has examined seasonal variation in CRP in patients with AS. That Turkish study showed, in contrast to our findings, the highest mean CRP values in winter, although without statistical significance [8]. Additionally, the authors reported a statistically significant variation in mean BASDAI scores and visual analogue scale assessment of pain, with both measures higher in winter compared to summer [8]. In our study, which had a substantially larger study population, we found a statistically significant seasonal variation in mean CRP values which varied similarly with other measures of disease activity, such as BASDAI and BASFI, but in contrast to the previous study we observed the highest disease activity in summer and beginning of fall. However, whether the difference in results are due to difference in climate between Turkey and Sweden or due to organizational factors is unknown.

The seasonal variation in CRP has previously been examined in healthy populations, but the findings have been somewhat contradictory. Some studies have shown variation throughout the year, while others report no significant seasonal differences [20–25]. In a South Korean study of 18 445 healthy individuals, excluding individuals with suspected acute inflammatory diseases, CRP was found to vary significantly over the year, with higher mean values in winter and spring compared to summer [24]. Similarly, a biobank study from the United Kingdom, which included 329 261 participants, showed a variation in CRP levels with peak values during winter [25]. These findings are not consistent with our results, which may indicate that the variation in CRP values observed in our study could be related to disease activity, organizational factors, that the seasonal variation in CRP might differ between populations or is influenced by climate.

One may hypothesize the general well-being vary throughout the year. To the best of our knowledge, no studies of seasonal variation in quality of life (QoL) and perceived well-being have been performed on a healthy population in Sweden. Since disease activity in axSpA is mainly assessed by self-reported outcome measures, possible underlying and unrelated seasonal variation in perceived well-being and QoL, unrelated to axSpA, could confound actual disease-related assessments. In the present study Eq. 5D scores were available, but no seasonal variation was found. Since no variation in Eq. 5D scores was seen, it is unlikely that our observed seasonal variation in patient reported outcomes is affected by an underlying seasonal variation in general QoL. The observed seasonal variation may therefore represent a true variation in disease activity or be an effect of priorities made when the resources are limited.

Previous studies regarding seasonal variation in inflammatory joint diseases have primarily been undertaken in individuals with RA. These studies, both conducted in Japan, have shown statistically significant variation in disease activity between seasons. In contrast to our findings, these studies found a higher disease activity in spring compared to fall [4, 5]. In line with our findings, the numerical variation between seasons was minimal. Notably, our study focuses on the seasonal variation in symptoms related to axial disease, which may limit the direct comparison with RA which predominantly affects peripheral joints.

In our study, axial mobility was lowest towards the end of the year and highest in spring, or in northern Sweden late winter immediately before onset of spring. This suggests that axial mobility may be more affected by trend in temperature rather than the absolute temperature or season. The association between weather conditions and different aspects of QoL, as well as axial mobility, has previously been examined in a study of 146 individuals with AS. In that study, physical QoL was rated higher in summer compared to winter and there was an association between higher spinal mobility and warmer temperatures [7]. Conversely, the authors report a lower perceived physical QoL associated to higher temperatures and wind speed four weeks prior to the completion of questionnaires. As such, the reported findings are contradictory, and further raise questions regarding the association between disease activity, axial mobility, and perceived QoL.

The strengths of the present study include the large study population with validated diagnoses and repeated measurements from individuals over an extended period, as well as the consideration of regional differences in terms of seasons. However, this study had some limitations that need to be further addressed. Data in the SRQ does not contain information on diseases other than axSpA, possibly introducing confounding factors, which should be considered when interpreting our results. However, our study population is relatively young, and therefore presumably healthy with few other diseases than axSpA, and since disease activity was measured with tools specific for axSpA the possible influence of unknown diseases or comorbidities is unlikely to significantly affect our results. Finally, since our data set is very large, confounding diseases would have to be extraordinarily common in the study population to entirely explain the results.

It is not certain the data used is representative for all individuals with axSpA, since the generalizability of data on individuals with axSpA from SRQ has not previously been examined. A large proportion of the study population has, or has had, biological treatment at some point during the period examined. A possible explanation to this is that individuals with axSpA and a consistent low disease activity treated in the primary care system are usually not included in SRQ and therefore not included in the present study. This suggests that our population may originally have had a disease activity above average, which could confound our findings and limit the generalizability of our results. Another confounding factor to consider is the number of visits tends to be lower during summer months, particularly in July, due to summer holidays. Consequently, the visits occurring during this period may involve patients with higher disease activity. Another important consideration is that variations in temperature, as well as other environmental factors, during seasons vary greatly between countries and geographical areas, which negatively affects the generalizability of the results of our study.

In summary, we have shown that parameters of disease activity, physical function and well-being in axSpA vary throughout the year. While the variation is statistically significant, it is numerically minimal, suggesting no clinical relevance. Whether the seasonal variation seen in our study represents a true variation in disease activity or is due to organizational factors, i.e. summer holidays, is undetermined. However, the highest mean values were found during the period of summer holidays indicating that organizational factors are important to consider while interpreting our results. Further studies are necessary to investigate whether the variation is caused by weather, trends in weather, or specific seasonal changes.

## Electronic supplementary material

Below is the link to the electronic supplementary material.

Supplementary Material 1

## Data Availability

Individual level data may not be made publicly available due to The General Data Protection Regulation (GDPR) and privacy concerns. The data used for this study contains protected health information. Data is available from Umeå University for researchers who meet the criteria for access to confidential data and have a data use agreement. Umeå University has restricted public sharing of data containing patient information. Inquiries for access should be addressed to the Department of Public Health and Clinical Medicine, Umeå University, Umeå, Sweden (info.folkhalsa@umu.se).
