# Supplementary material for: Minimal seasonal variation in disease parameters of axial spondyloarthritis: a register-based study
Source: Rheumatol Int. 2025 Jul 17;45(8):174. doi: 10.1007/s00296-025-05913-4 (PMC12271298; doi:10.1007/s00296-025-05913-4)
Supplement: Supplementary file 1 — Supplementary Material 1 [file 296_2025_5913_MOESM1_ESM.docx]

**Supplementary material**

**
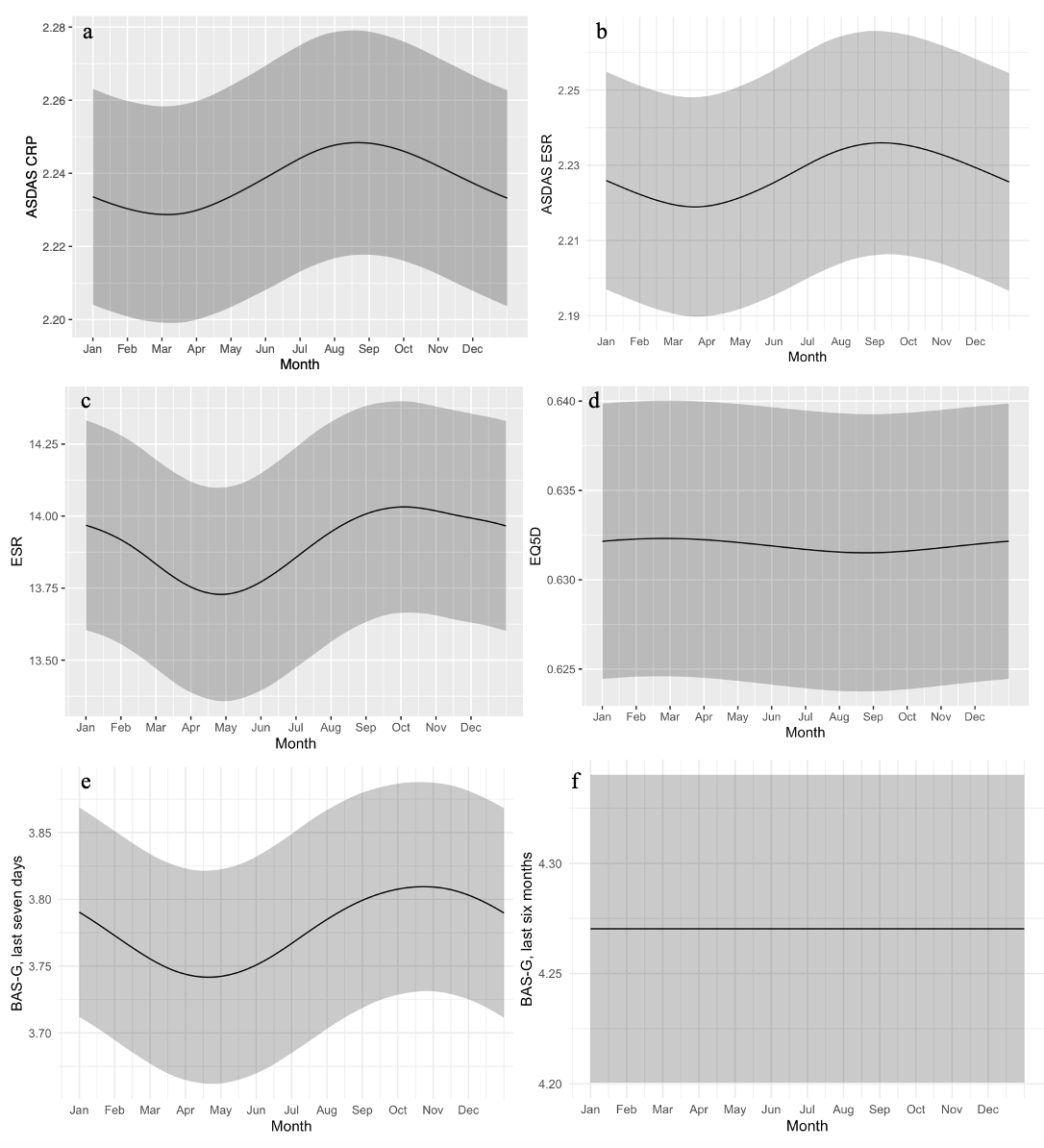
Supplementary fig. 1** Temporal variation, for the entire country, in mean values with splines. Figure a displaying ASDAS CRP (p = 0.1), figure b displaying ASDAS SR (p = 0.1), figure c displaying ESR (p = 0.52), figure d displaying EQ5D (p = 0.3), figure e displaying BAS-G, last 7 days, (p = 0.06) and figure f displaying BAS-G, last 6 months, (p = 1). The main line represents the smoothed mean curve, while the ribbon signifies the 95% confidence interval

BASDAI: Bath Ankylosing Spondylitis Disease Activity Index. BASFI: Bath Ankylosing Spondylitis Functional Index. BASMI: Bath Ankylosing Spondylitis Metrology Index. BAS-G: Bath Ankylosing Spondylitis Global Score. CRP: C-reactive protein. ESR: Erythrocyte sedimentation rate. ASDAS: Ankylosing Spondylitis Disease Activity Score

**Supplementary fig. 2** Median values of CRP, BASFI, BASDAI, BASMI, and EQ5D split by seasons and gender. The boxes represent the interquartile range, with the line inside indicating the median. The whiskers extend to the 10th and 90th percentiles

BASDAI: Bath Ankylosing Spondylitis Disease Activity Index. BASFI: Bath Ankylosing Spondylitis Functional Index. BASMI: Bath Ankylosing Spondylitis Metrology Index. CRP: C-reactive protein

**Supplementary fig. 3** Temporal variation, for individuals with a clinically relevant variation (i.e. >1 cm, >0.7 cm, >30%) between highest and lowest value in BASDAI (fig. a, n = 24 401), BASFI (fig. b, n = 21 545), and BASMI (fig. c, n = 2770) in mean values with splines (p 0.1). The main line represents the smoothed mean curve, while the ribbon signifies the 95% confidence interval

BASDAI: Bath Ankylosing Spondylitis Disease Activity Index. BASFI: Bath Ankylosing Spondylitis Functional Index. BASMI: Bath Ankylosing Spondylitis Metrology Index
